# Quality of Life (QoL) among medical students in Tunisia: a study using the WHOQOL-BREF instrument

**DOI:** 10.1192/j.eurpsy.2023.770

**Published:** 2023-07-19

**Authors:** D. Njah, M. Lagha, S. Boudriga, W. Homri, I. Ben Romdhane, R. Labbene

**Affiliations:** Psychiatry “C”, Razi hospital, tunis, Tunisia

## Abstract

**Introduction:**

Mental health problems such as stress, anxiety and depression have been described among medical students and are associated with poor academic and professional performance. That’s why having a satisfying quality of life (QoL) is one of the main sources of motivation for students for their future.

**Objectives:**

Our objectives were to assess the QoL of medical students and residents in Tunisia and to explore the influencing factors on this one.

**Methods:**

This was a cross-sectional study among medical students and residents in Tunisia, all universities included, where they completed a questionnaire which comprised the WHOQOL-BREF instrument in its french version and several socio-demographics questions, in September 2022. Statistical analysis was performed by SPSS 26.0.

**Results:**

One hundred twenty-five medical students and residents were included in our study. The mean age was 26.10(±3.41) years and most of them were female (73%). Mean scores of the WHOQOL-BREF in the physical, psychological, social and environmental domains were 36.51 (±11.54), 45.22 (±15.71), 37.19 (±18.61) and 52.94 (±14.84), respectively. Students and residents had a relatively higher environmental mean score and a lower physical health mean score. The lowest mean score of the physical domain was observed in the 6th year students while the lowest mean scores of the psychological, social and environmental domains were observed in the medical students. Besides, we found a higher score of social and environmental domains in the residents group. In addition, we found a high correlation between psychological and environmental domains (p=0.000), psychological and social domains (p=0.021). We also found a correlation between age and social domain (p=0.034), in fact, the higher the age was the better the score of the social domain was. And finally we found a significant relationship between the environmental domain and the level of studies (p=0.05).

**Conclusions:**

Physical health, psychological, social and environmental issues have an important impact on the QoL of our population and hence their future. Certain factors seem to be involved and have to be taken into consideration in order to improve QoL among medical students and residents.

**Disclosure of Interest:**

None Declared

